# Medico-Legal and Medico-Ethical

**Published:** 1892-05

**Authors:** 


### MEDICO-LEGAL AND MEDICO-ETHICAL.

### DENHOLM VS. TAIT.

This case came before Mr. Justice Collins at the Manchester assizes last week. Mr. Gully, Q. C., M. P., Mr. Bingham, Q. C., and Mr. Sutton were for the plaintiff; Mr. Kennedy, Q. C., and Mr. Ashton for the defendant. Mr. Gully said that the plaintiff in this action was Dr. Denholm, a physician and surgeon practising in Manchester for many years past. The defendant, Mr. Lawson Tait, was an eminent surgeon practising at Birmingham. He was a specialist and an operator. The action was one for libel.

The statement of the counsel and the evidence of the plaintiff showed that the plaintiff had been suffering for some years from uterine myoma, which, in 1889, began to cause inconvenience. Dr. Lloyd Roberts and Mr. Whitehead were consulted by Dr. Denholm, under whose care the patient had been for some years, and recommended the use of Apostoli's electrical treatment. That treatment was continued for some time, but without effect, and in the course of the treatment an accident occurred in the operation, which gave rise to a vesical fistula. This fistula was twice operated upon, but each time ineffectually. In December, 1891, the patient, Mrs. Payne, consulted Mr. Tait, who, it was alleged, blamed the electrical proceeding, and advised removal of the tumor. According to the statement of Mr. Gully, Q. C., he advised an operation, and said the risk was very small indeed,—that he only lost one case in sixty. Mrs. Payne asked Mr. Tait particularly about the fistula, and he said that he could put the matter right. Mr. Tait told Mrs. Payne's husband that the risk attending the operation might be put at five per cent. at the outside. The lady underwent the operation, and Mr. Gully suggested, for reasons which he stated, that the lady was not at the time in proper condition of health to be operated upon. Soon after the operation the husband called, and was told the patient was going on splendidly, but within forty-eight hours he got a telegram summoning him at once to his wife, who was much worse. Half an hour after he arrived she died, without having recognized him. The husband was anxious to see Mr. Tait, but was unable to do so. The following letter, which contained the alleged libel, was handed to him:

MY DEAR SIR.—I saw your wife, with Mr. Christopher Martin, between two and three this morning, and matters were going on very fairly with her. At 7.30 I was summoned again, and found her sinking. You were summoned at once, and, I am glad to learn, reached her bedside before she died. The immediate cause of the unfortunate result is the rupture of a blood-vessel at or near the spot where the electrical needles caused so much damaging inflammation and sloughing. Had she never been submitted to that treatment, the case would have been a straightforward one, and her recovery almost certain.

Whether privileged or not, which they would have to consider, that meant that death was owing to the fact that her previous medical attendant had submitted her to the electrical treatment. Mr. Payne naturally considered this letter as imputing that the death of his wife was entirely owing to improper medical treatment before the operation, and when he got back to Manchester he immediately went to see Dr. Denholm, to whom he showed the letter. The plaintiff took the letter as containing a charge against himself, and told Mr. Payne the charge was entirely unfounded and untrue, that hemorrhage was not the cause of death, and that the best way to find out the cause of death was a *post mortem* examination, which was afterwards carried out by three eminent medical men, the result of which was to show that death was not due to hemorrhage, or a rupture of a blood-vessel, or to damaging inflammation and sloughing consequent upon the previous electrical treatment. What it did show was that death had resulted from peritonitis, the result of the operation. Mr. Gully then detailed the condition of the patient after the operation, and gave her temperature, pulse, and treatment up to her death.

Witnesses were called to show, as the result of *post mortem* examination, that the cause of death was not hemorrhage as suggested, but peritonitis. It appeared, however, that the *post mortem* examination had been made by gaslight in the coffin, and that the hematocele of the broad ligament had been overlooked, while the evidence of peritonitis was very slight.

Mr. Kennedy, in his statement for the defense, pointed out that it was admitted by Mr. Gully that Mr. Tait was entitled and bound to make a truthful report to the husband of the lady as to what he considered to be the facts of the case and the causes of unsuccess. The letter, if honestly written, as lawyers knew, was written on a privileged occasion. It was a letter on the part of Mr. Tait which legally, morally, and socially he was entitled to write. The writer wrote the facts, as he believed, of the case, but the other side alleged that that statement was erroneous in fact, and that it reflected upon the treatment which the patient had received from others. It was admitted that so long as Mr. Tait wrote what he honestly believed to be the facts, his letter was privileged. If it were otherwise, life would be intolerable. It would be impossible that life could be tolerated if the truth might not be stated, however unpleasant to others, on privileged occasions. Where, however, a person said a thing, not because he believed it, but to do someone an injury, then the law properly stepped in and made the party responsible, and the privilege no longer existed. In this case the whole evidence of the other side had been directed to show that the statement in the letter written by Mr. Tait was not merely erroneous as regarded the cause of death, but that it was a statement which Mr. Tait could not have honestly believed to be the truth. That his client denied. Mr. Kennedy then detailed the circumstances of the case, and proceeded to call evidence.

Mr. Lawson Tait was then called. He gave the notes of his conversation and of the examination of the patient. The myoma was a large one, and a tumor which had grown in seven years to that size would reach a fatal size before change of life. There was a history of dysmenorrhea, and the failure of the electrolysis; the production of the fistula, in the cure of which two distinguished surgeons, Mr. Whitehead and Dr. Lloyd Roberts, had failed, was absolutely unique in his experience. He was perfectly certain that the fistula could not be cured until the tumor was removed. If he had said anything at all about percentages, he could only have said, in the communication to Mr. Payne, that the failures were from eight to ten per cent. There was a history of inflammatory symptoms after electrical operation. In the operation, the tumor was found to be very adherent to the peritoneum, as the result of inflammation, and the operation took over an hour. He maintained his opinion as expressed in the letter, and he had nothing to withdraw.

Mr. Gully, on behalf of the plaintiff, and without calling the witnesses of Mr. Tait, said that having heard the statement of the defendant, his client in the fullest manner withdrew the imputation that Mr. Tait had wilfully or recklessly misstated the cause of death; and Mr. Walter Whitehead, who had stated in the box that in his opinion Mr. Tait had recklessly operated on the patient, now having heard Mr. Tait, wished to withdraw that statement.

Mr. Kennedy said that he concurred with that view, and accepted the full and handsome withdrawal of any imputations against Mr. Tait's character, and he (Mr. Kennedy) could only repeat what he had said in the beginning, and what Mr. Tait had said in his letter, that he had no intention whatever of reflecting upon the character of the medical gentleman who had attended Mrs. Payne.

The Judge said he was exceedingly pleased at the close of the case. He could not help feeling that if the parties had had the opportunity of meeting and knowing the facts of the case on both sides, these difficulties would never have arisen between them.

Mr. Tait said he fully accepted Mr. Walter Whitehead's withdrawal.

---

It is reported that the combined obstetrical experience of Barker, Taylor, and Thomas, of New York, Penrose and Wallace, of Philadelphia, and Chatard, of Baltimore, covers a period of two and a quarter centuries, during which they have attended at the births of over forty-five thousand children, and they have been compelled to perform the operation of craniotomy less than twenty times.—*Toledo Medical Compend.*

